# Integrated transcriptomic and metabolomic analyses reveal the molecular mechanisms of sugar accumulation and yield trade-off in sugar beet

**DOI:** 10.3389/fpls.2026.1874591

**Published:** 2026-09-01

**Authors:** Xiaoshan Bai, Sizhong Li, Abudukadier Kuerban, Hong Sha, Daoqian Yu, Xinjiu Dong, Jing Zhu, Xiaojing Liu, Jipeng Zhang, Ziming Wang, Zhengxing Liu, Liya Lv, Teng Ma, Liang Meng, Hengyu Pan, Huajun Liu, Zhimin Wang

**Affiliations:** 1 Crop Research Institute, Xinjiang Uygur Autonomous Region Academy of Agricultural Sciences, Urumqi, China; 2 Xinjiang Sugar Beet Industry Engineering Technology Research Center, Urumqi, China; 3 Aksu Regional Agricultural Technology Extension Center, Xinjiang Uygur Autonomous Region, Aksu, China; 4 Xinjiang Uygur Autonomous Region Seed Industry Development Center (Xinjiang Uygur Autonomous Region Hainan Improved Seed Breeding Station), Sanya, China; 5 Xinjiang Daziran Biotechnology Co., Ltd., Kashgar, China; 6 College of Agronomy and Biotechnology, China Agricultural University, Beijing, China

**Keywords:** carbon partitioning, metabolism suppression, sugar beet, transcription factor hub, yield-sugar trade-off

## Abstract

**Background:**

The negative correlation between taproot yield and sugar content remains a persistent challenge in sugar beet (*Beta vulgaris* L.) breeding. This trade-off reflects the competitive partitioning of finite assimilated carbon between structural growth and vacuolar storage. However, the transcriptional and metabolic mechanisms underlying this shift remain poorly characterized.

**Methods:**

We integrated transcriptomic and widely-targeted metabolomic data across five developmental stages of taproots from two lines with contrasting phenotypes: a high-sugar/low-yield line (Group K) and a low-sugar/high-yield line (Group D).

**Results:**

Our analysis revealed that high sucrose accumulation is driven by minimized metabolic consumption rather than enhanced biosynthesis. While the high-yield line (Group D) sustained elevated expression of sucrose synthesis genes (*SPS*, *SPP*) alongside elevated expression through the tricarboxylic acid (TCA) cycle (*CS*, *ACLY*, *MDH2*) and nitrogen assimilation (*GLUL*, *GAD*) to fuel continuous cambial expansion, the high-sugar line (Group K) showed progressive attenuation of these primary metabolic pathways during maturation. Consequently, Group K exhibited depleted pools of citrate, malate, and glutamine, but highly accumulated sucrose and osmoprotectants like trehalose and raffinose. Weighted Gene Co-expression Network Analysis (WGCNA) identified a core co-expression module strongly correlated with the high-sugar trait. Within this module, transcription factors AP2, bHLH78, REM16, SRS3, and TIFY4b were identified as hub genes, suggesting possible regulatory roles in the suppression of growth-associated carbon consumption.

**Conclusions:**

The yield-sugar trade-off in sugar beet appears to reflect differential carbon allocation between primary metabolic pathways and sucrose storage, associated with distinct transcriptional programs in the two lines. The identified transcription factor hubs may participate in regulating the balance between growth-associated carbon use and storage metabolism. These findings provide candidate molecular targets for future studies aimed at simultaneously improving yield and sugar content in sugar beet.

## Introduction

In higher plants, the partitioning of photosynthetically fixed carbohydrates between source tissues (e.g., mature leaves) and sink tissues (e.g., roots, fruits, and seeds) is a fundamental biological process that governs growth, development, yield, and quality (Zhao et al., 2022). At the core of this source-sink system are the synthesis of photoassimilates in source tissues, their long-distance transport via the phloem, and their subsequent unloading, metabolism, and storage in sink tissues (Rosado-Souza et al., 2023). Sugar beet (*Beta vulgaris* L.), as one of the most important sugar crops in temperate regions worldwide, supplies approximately 30% of the global sugar output. Its fleshy taproot is capable of accumulating sucrose, its primary storage compound, to remarkable levels, often constituting 15-20% of the root’s fresh weight (Zhang et al., 2017). Consequently, sugar beet is not only of considerable agro-economic importance but also serves as a valuable model system for investigating the molecular mechanisms of carbohydrate transport, sink organ development, and solute compartmentalization.

The unique anatomy of the sugar beet taproot underlies its sucrose storage capacity. During development, anomalous cambial activity gives rise to multiple concentric rings of vascular bundles and parenchyma tissue. This supernumerary growth pattern expands the tissue area available for sucrose unloading and storage, thereby determining the final biomass of the root (Jammer et al., 2020). At the physiological and biochemical levels, sucrose accumulation involves sequential steps of phloem unloading, metabolism, and vacuolar storage. Upon transport through the phloem, sucrose is unloaded into the apoplast or symplast of the root parenchyma (Lemoine et al., 2013). In the early stages of root development, high acid invertase activity hydrolyzes sucrose into hexoses, which provide the energy and carbon skeletons required for cell division and differentiation (Godt and Roitsch, 2006). As the root transitions into a phase of rapid expansion and sugar accumulation, the activity of acid invertase is suppressed while that of sucrose synthase becomes predominant, favoring the direct utilization or storage of sucrose (Fugate et al., 2019). Sucrose is then actively transported into the vacuole against a concentration gradient by tonoplast transporters, notably BvTST2.1, in a process driven by the proton motive force of the vacuolar H^+^-ATPase and H^+^-Ppase (Jung et al., 2015).

However, a long-standing challenge in sugar beet genetic improvement and breeding is the significant negative correlation commonly observed between root yield (determined by biomass) and sugar content (Hassani et al., 2024). This trade-off means that high-yielding varieties tend to have lower sugar content, whereas high-sugar varieties often exhibit compromised yields. This antagonism reflects a competition for photoassimilates between two metabolic outcomes: structural growth and vacuolar sucrose storage (Jammer et al., 2020). On one hand, root expansion and biomass gain depend on continuous cell proliferation and enlargement (Jammer et al., 2020). This process requires substantial carbon allocation to primary metabolism, including respiratory consumption via glycolysis and the tricarboxylic acid (TCA) cycle to generate ATP and reducing equivalents, as well as biosynthesis of macromolecules such as proteins and nucleic acids (Gippert et al., 2022). On the other hand, sucrose accumulation requires the downregulation of structural metabolism and reduction of respiratory carbon loss at specific developmental stages (Fugate et al., 2024), redirecting carbon flux toward the vacuolar sucrose pool.

At the molecular level, several key components of sucrose metabolism in sugar beet have been characterized. The tonoplast sucrose transporter BvTST2.1 has been identified as the primary mediator of vacuolar sucrose compartmentalization, with its expression and activity closely linked to taproot sugar content (Jung et al., 2015). The developmental regulation of key enzymatic steps—including sucrose phosphate synthase (*SPS*), sucrose synthase, and acid invertase—has been described in relation to the growth-to-storage transition (Godt and Roitsch, 2006; Fugate et al., 2019). Transcriptomic profiling of taproot development in a single genotype has revealed stage-dependent gene expression programs associated with sucrose accumulation (Zhang et al., 2017), while recent metabolomic and multi-omics studies have characterized carbon and nitrogen metabolism during storage conditions and abiotic stress (Gippert et al., 2022; Gutschker et al., 2022). However, these studies have generally been conducted within a single genetic background or under specific environmental conditions and have not directly compared lines with divergent yield-sugar phenotypes across the full developmental trajectory. Consequently, the transcriptional regulatory networks that govern the shift from growth-oriented carbon metabolism to sucrose storage—and the gene modules underlying the yield-sugar trade-off—remain poorly defined.

In particular, the regulatory factors and co-expressed gene networks underlying this metabolic transition remain to be identified. This represents a key limitation in efforts to simultaneously improve yield and sugar content in sugar beet breeding. High-throughput transcriptomics and metabolomics offer approaches for dissecting the molecular basis of such complex agronomic traits. Comparative multi-omics analysis across developmental stages and contrasting genetic backgrounds can help identify co-expressed gene modules, metabolic markers, and candidate regulators associated with the yield-sugar divergence (Gutschker et al., 2022).

In this study, we integrated transcriptomic and metabolomic profiles of two sugar beet lines with contrasting phenotypes—a high-sugar/low-yield line (S72-8) and a low-sugar/high-yield line (DR41K)—across five developmental stages of taproot growth. Our objective was to characterize the transcriptional and metabolic programs associated with the high-sugar phenotype and to identify co-expressed gene modules and candidate regulatory factors underlying the yield-sugar divergence between the two lines. We hypothesized that the phenotypic divergence between these two lines reflects distinct developmental transcriptional programs that differentially allocate carbon between primary metabolic pathways and vacuolar sucrose storage. The findings are intended to provide candidate gene targets for the genetic improvement of sugar beet with respect to both yield and sugar content.

## Materials and methods

### Plant materials and field experimental design

Two sugar beet (*Beta vulgaris* L.) inbred lines with divergent root yield and sugar content phenotypes were used: the high-sugar/low-yield line S72-8 (Group K) and the low-sugar/high-yield line DR41K (Group D). Both lines were developed by the Sugar Beet Innovation Team of the Xinjiang Academy of Agricultural Sciences through multi-generation self-pollination under individual plant isolation. Line selection was based on a two-year replicated field evaluation (2023–2024) of 31 inbred lines conducted at the Manas Experimental Station, Xinjiang, China. Across both years, S72–8 consistently ranked first in root sugar content among all 31 lines (two-year mean: 15.50%), while DR41K ranked last (two-year mean: 12.45%), with a difference of 3.05 percentage points. In terms of root yield, the two lines showed contrasting trends: DR41K ranked 15th (97.2 t/ha) and S72–8 ranked 21st (91.0 t/ha) across the two-year mean. The phenotypic divergence in sugar content between the two lines was stable across years and statistically significant (one-way ANOVA, p < 0.05), supporting their selection as contrasting materials for subsequent molecular analysis.

Field trials were conducted in 2024 at the Agricultural Experiment Station in Lanzhouwan Township, Manas County, Xinjiang, China. The experiment followed a randomized block design with three biological replicates per line. Each plot (7 m²) consisted of two rows (7 m × 1 m) with row spacing of 50 cm and plant spacing of 17.5 cm. Sowing occurred on April 22nd, with full emergence by April 30th. Uniform agronomic management—including mechanized direct seeding, pressurized drip irrigation under plastic mulch, and consistent fertigation—was applied across all plots. Final harvest occurred on October 10th.

### Tissue sampling and phenotypic measurements

Five developmental stages were sampled: July 22nd (rapid sugar accumulation initiation, Stage 1, ~83 days after emergence), August 11th (Stage 4, ~103 days, active sugar accumulation), August 31st (Stage 6, ~123 days, accelerated sucrose storage), September 20th (Stage 8, ~143 days, onset of leaf senescence), and October 10th (harvest, Stage 9, ~163 days, harvest maturity). Stage numbering follows the developmental classification of Jammer et al. (2020). At each time point, 5 representative plants per biological replicate were selected. Taproot tissues were sampled using a custom-made stainless-steel core sampler (8–10 mm diameter) inserted diagonally at 45° to include representative periderm, phloem, cambial rings, and xylem parenchyma. Samples were flash-frozen in liquid nitrogen and stored at -80 °C.

At harvest, agronomic traits measured included functional leaf number, shoot and root fresh and dry weights, plot yield (converted to t/ha), and root sugar content determined by polarimetry. Differences between lines were assessed using Student’s t-test; p < 0.05 was considered statistically significant.

### Total RNA extraction, library construction, and high-throughput transcriptome sequencing

Total RNA was extracted from frozen root tissue, which was first ground to a fine powder in liquid nitrogen, using an RNAprep Pure Plant Plus Kit (Tiangen, Beijing, China) according to the manufacturer’s protocol. RNA purity was assessed using a NanoDrop 2000 spectrophotometer (Thermo Fisher Scientific, USA). RNA integrity and concentration were precisely quantified using an Agilent 2100 Bioanalyzer system with an RNA Nano 6000 Assay Kit (Agilent Technologies, USA). Only samples with an RNA Integrity Number (RIN) ≥ 7.0 were used for sequencing.

For library construction, mRNA was purified from total RNA using oligo(dT)-attached magnetic beads. The purified mRNA was fragmented into short pieces using a fragmentation buffer. First-strand cDNA was synthesized using random hexamer primers and M-MuLV reverse transcriptase, followed by second-strand cDNA synthesis using DNA Polymerase I and RNase H. The resulting cDNA fragments were subjected to end-repair, A-tailing, and ligation with sequencing adapters. Fragments of a specific length were selected using the AMPure XP system (Beckman Coulter, USA), and the final sequencing libraries were generated by PCR amplification. After quality control, the libraries were sequenced on an Illumina NovaSeq 6000 platform using a paired-end 150 bp (PE150) strategy. A total of 30 libraries (2 lines × 5 time points × 3 replicates) were constructed and sequenced.

### Widely-targeted metabolomics sample preparation and LC-MS/MS analysis

Widely-targeted metabolomics analysis of root tissues was performed in collaboration with Metware Biotechnology Co., Ltd. (Wuhan, China). Samples stored at -80 °C were lyophilized and ground to a powder using a grinder (MM 400, Retsch, 30 Hz, 1.5 min). A 100 mg aliquot of the lyophilized powder was extracted with 1.2 mL of pre-chilled 70% aqueous methanol containing L-2-chlorophenylalanine as an internal standard. The mixture was vortexed for 30 s, sonicated in an ice bath for 30 min, and then centrifuged at 12,000 × g for 10 min at 4 °C. The supernatant was collected, passed through a 0.22 μm microporous filter, and transferred to a sample vial for LC-MS/MS analysis.

Metabolite data acquisition was performed on a UPLC-MS/MS platform consisting of an ultra-performance liquid chromatograph (UPLC, Shimadzu Nexera X2) coupled to a tandem mass spectrometer (MS/MS, Applied Biosystems 4500 Q TRAP). Chromatographic separation was achieved using a Agilent SB-C18 column (1.8 µm, 2.1 mm × 100 mm). The mobile phase consisted of solvent A (ultrapure water with 0.1% formic acid) and solvent B (acetonitrile with 0.1% formic acid). The gradient elution program was as follows: 0–2 min, 5% B; 2–10 min, linear increase from 5% to 95% B; hold at 95% B until 11 min; decrease to 5% B in 0.1 min and equilibrate for 1.9 min. The flow rate was 0.35 mL/min, the column temperature was 40 °C, and the injection volume was 2 μL.

The mass spectrometer was operated with an electrospray ionization (ESI) source at a temperature of 550 °C. The ion spray voltage was set to +5500 V in positive ion mode and -4500 V in negative ion mode. The curtain gas (CUR), collision gas (CAD), and nebulizer gas (GS1, GS2) were set to 25 psi, Medium, and 55/60 psi, respectively. Precursor and fragment ion data were acquired using the multiple reaction monitoring (MRM) mode. Data processing, including baseline filtering, peak identification, integration, and retention time correction, was performed using Analyst software (v1.6, Framingham, MA, USA). Metabolite identification was conducted by matching against an in-house database (MWDB) and public databases.

### Transcriptome and metabolome data normalization and differential analysis

For transcriptomic data, raw reads were first processed using fastp (Chen et al., 2018) to remove adapters and low-quality sequences, yielding clean reads. The high-quality reads were then aligned to the sugar beet reference genome using HISAT2 (Kim et al., 2019). The number of reads mapped to each gene was counted with featureCounts (Liao et al., 2014), and gene expression levels were quantified and normalized using the Fragments Per Kilobase of transcript per Million mapped reads (FPKM) method. Differentially expressed genes (DEGs) were identified using DESeq2 (Love et al., 2014). The thresholds for significant differential expression were set at |log2FoldChange| ≥ 1 and an adjusted p-value (FDR) < 0.05.

For metabolomic data, the metabolite abundance matrix was first log2-transformed and mean-centered. Multivariate statistical analyses, including principal component analysis (PCA) and partial least squares-discriminant analysis (PLS-DA), were performed using the ropls (Thévenot et al., 2015) package in R. Differentially abundant metabolites (DAMs) were identified based on a combination of multivariate and univariate statistics, with the criteria set as a Variable Importance in Projection (VIP) score ≥ 1 from the PLS-DA model and a p-value < 0.05 from an independent samples t-test.

### Time-series expression trajectory clustering and pathway enrichment analysis

To identify temporal gene expression patterns, a soft clustering analysis was performed on the filtered DEG matrix using the fuzzy c-means algorithm implemented in the Mfuzz R package (Kumar and Futschik, 2007). Prior to clustering, FPKM values for each gene were standardized to have a mean of 0 and a standard deviation of 1 (z-score normalization). The fuzzifier parameter (m) was set to its default value, and the optimal number of clusters (c) was determined by identifying the elbow point in a plot of the within-cluster sum of squared errors (WSS). For specific gene sets and module intersections, Kyoto Encyclopedia of Genes and Genomes (KEGG) pathway enrichment analysis was conducted using the clusterProfiler (Yu et al., 2012) R package based on a hypergeometric test. The resulting p-values were corrected for multiple testing using the Benjamini-Hochberg method, and pathways with a q-value < 0.01 were considered significantly enriched.

### Weighted gene co-expression network analysis and hub gene identification

To elucidate genome-wide co-expression modules and their association with phenotypic traits, a regulatory network was constructed using the WGCNA (Langfelder and Horvath, 2008) R package. To reduce noise, only genes with an FPKM ≥ 1 in at least 15 samples were included in the analysis. The pickSoftThreshold function was used to select the optimal soft-thresholding power (β =6), which satisfied the criterion for a scale-free topology (fit index R² > 0.8). A topological overlap matrix (TOM) was generated, and gene co-expression modules were identified using the dynamic tree cut algorithm with the following parameters: minimum module size (minModuleSize) = 30 and merge cut height for similar modules (mergeCutHeight) = 0.2. The module eigengene (ME) for each module was correlated with phenotypic data using Pearson correlation coefficients to identify key trait-associated modules. Intramodular connectivity (kME) was calculated to identify hub genes within these key modules. The co-expression network of core modules, using gene pairs with a weight > 0.4, was visualized using Cytoscape (Shannon et al., 2003).

### Real-time quantitative PCR validation

To validate the reliability of the RNA-seq data, qRT-PCR was performed on key DEGs involved in carbon-nitrogen metabolism and regulatory networks. Gene-specific primers were designed using Primer Premier 5.0 software based on their CDS sequences (primer sequences are listed in **Supplementary Table 1**). First-strand cDNA was synthesized from an equalized pool of total RNA using the Tiangen FastKing RT Kit. The qRT-PCR reactions were performed on a Applied Biosystems QuantStudio 6 Flex Real-Time PCR System. The 20 μL reaction mixture contained 10 μL of 2× SYBR Green Master Mix, 0.8 μL of each forward and reverse primer (10 μM), 2 μL of diluted cDNA template, and 6.4 μL of ddH_2_O. The thermal cycling program was: 95 °C for 3 min, followed by 40 cycles of 95 °C for 10 s and 60 °C for 30 s. The sugar beet Tubulin gene, *BvTUB* was used as the internal reference gene. Relative gene expression levels were calculated using the 2^-ΔΔCt^ method (Livak and Schmittgen, 2001). All reactions were conducted with three biological replicates and three technical replicates.

## Results

### Phenotypic characterization of the two contrasting lines

At harvest, agronomic traits were measured from five randomly selected plants per biological replicate, with three biological replicates per line, consistent with the sampling design of the omics experiment. S72-8 (Group K) exhibited significantly higher root sugar content (17.99 ± 1.11%) than DR41K (Group D; 14.84 ± 0.21%; p < 0.05, Student’s t-test). Conversely, DR41K showed significantly greater root fresh weight yield (137.5 ± 8.1 t/ha) compared to S72-8 (116.7 ± 5.3 t/ha; p < 0.05). These results confirm a clear negative relationship between root yield and sugar content between the two lines, consistent with the yield-sugar trade-off documented in the two-year selection trial and providing phenotypic justification for their use in subsequent transcriptomic and metabolomic analyses (**Supplementary Table 2**).

### Transcriptome sequencing and spatiotemporal dynamics of global gene expression

To characterize transcriptional changes during sugar beet root development, we performed high-throughput RNA sequencing on taproot tissues from the high-sugar line S72-8 (Group K) and the low-sugar line DR41K (Group D) across five consecutive developmental stages (stages 1, 4, 6, 8, and 9). After quality filtering, the 30 libraries yielded a total of 207.16 Gb of high-quality clean data. The number of total reads per sample ranged from 41,249,302 to 53,199,018, and the mapping rates to the sugar beet reference genome were consistently high, ranging from 91.07% to 92.61% (**Supplementary Table 3**). The GC content across all libraries was stable (42.15% to 42.88%), and the Q30 quality scores were all above 98.26%. The low intra-group coefficients of variation confirmed the reliability of the sequencing data for subsequent gene expression quantification.

**Figure 1:**
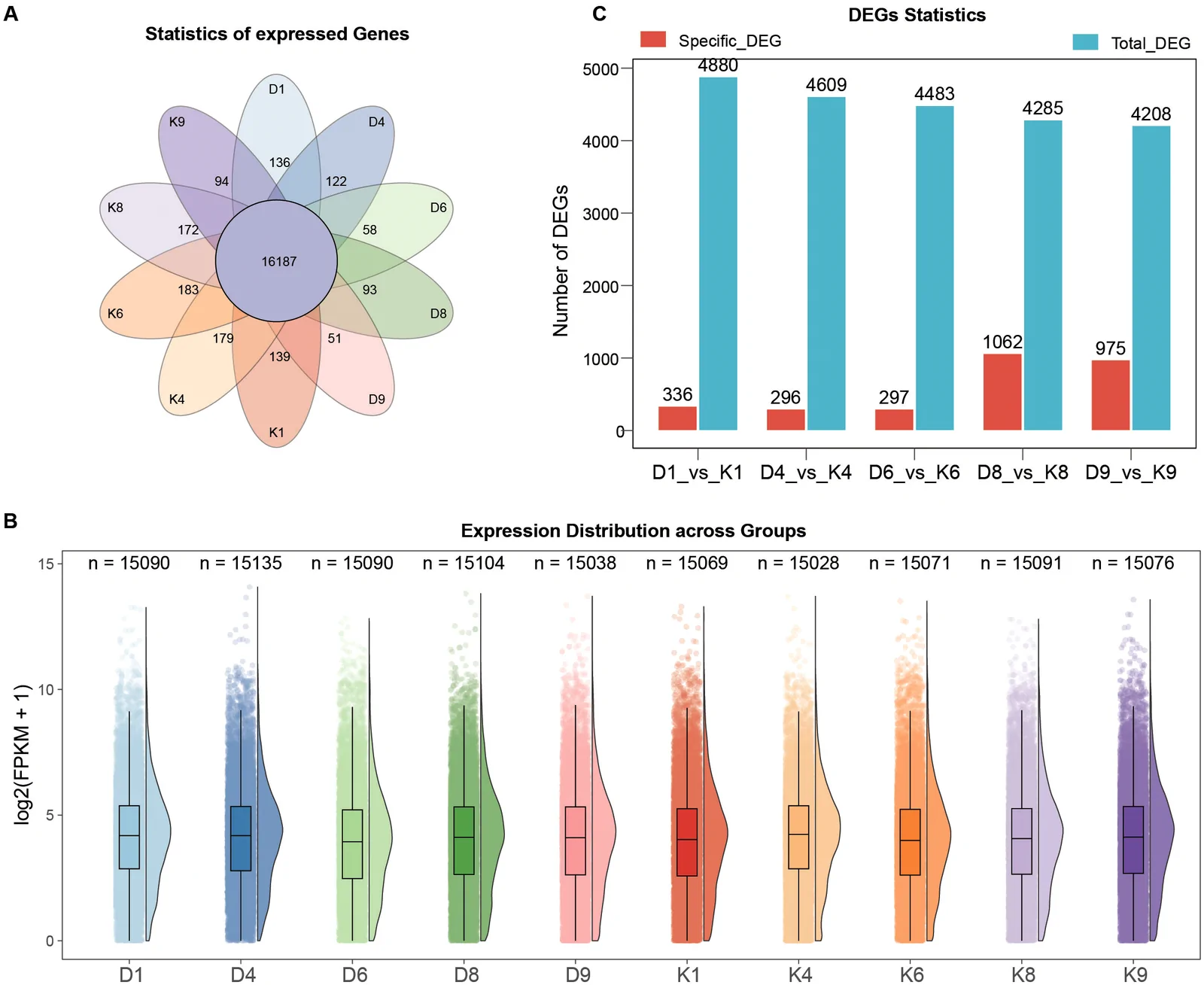
Transcriptional divergence between contrasting carbon allocation strategies in sugar beet taproots. **(A)** Venn diagram showing the distribution of expressed genes (FPKM ≥ 1) across ten developmental samples (D1-D9: Group D; K1-K9: Group K). The central intersection represents 16,187 core transcripts shared across all samples. **(B)** Violin plots showing the distribution of gene expression levels ([og2(FPKM + 1)] across samples, demonstrating consistent quantification. **(C)** Dynamics of differentially expressed genes (DEGs) between lines at corresponding stages, showing amplification of transcriptional divergence during late developmental stages (S6-S9).

A Venn diagram analysis of expressed genes revealed a core set of 16,187 transcripts shared across all samples, accounting for 60.34% of the total expressed genes, indicating a conserved transcriptional core alongside line- and stage-specific expression patterns (**Figure 1A**). Violin plots showed consistent median and distribution of expression levels across all samples with no systematic biases, confirming quantification stability (**Figure 1B**). Comparison of DEGs between the two lines at each stage showed that transcriptional divergence increased progressively during development. Specifically, in a stage-by-stage comparison between Group D and Group K, the divergence in their expression profiles widened in later stages, with the number of line-specific DEGs increasing to 1,062 and 975, respectively (**Figure 1C**). Concurrently, the total number of DEGs detected in these comparisons decreased to 4,285 and 4,208. This suggests that transcriptional divergence between the two lines became more pronounced during the rapid sugar accumulation phase.

### Metabolic phenotype reconstruction and carbohydrate homeostasis during root development

**Figure 2:**
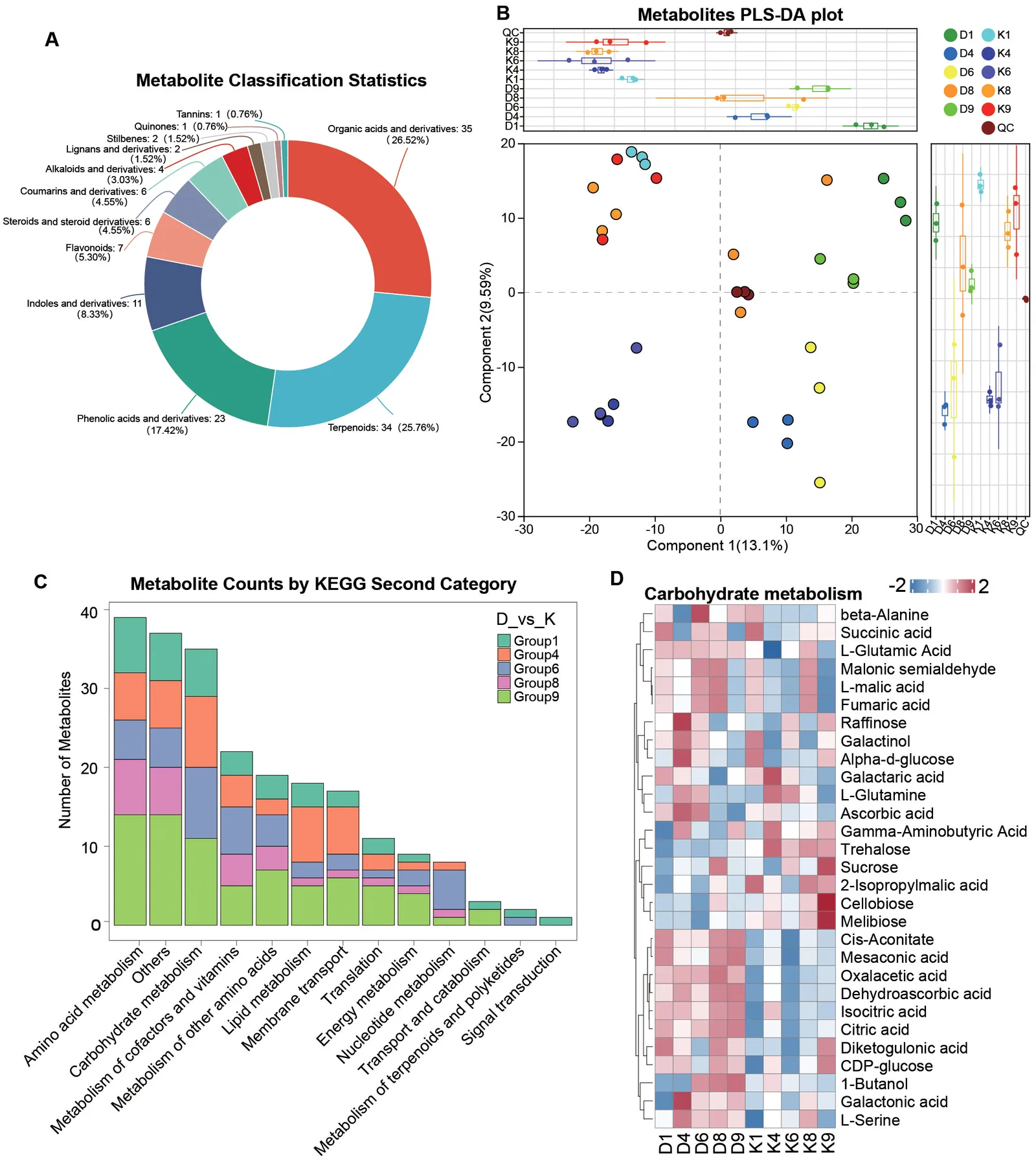
Metabolomic reconstruction reveals divergent carbon utilization strategies. **(A)** Classification of 568 detected metabolites. **(B)** PLS-DA score plot showing orthogonal separation by genotype (Component 1) and developmental trajectory (Component 2). **(C)** KEGG pathway distribution of differentially abundant metabolites (DAMs) across developmental stages. **(D)** Heatmap of carbohydrate metabolism DAMs showing Group K’s accumulation of sucrose and osmoprotectants (trehalose, raffinose) versus Group D’s maintenance of TCA cycle intermediates (citrate, malate, oxaloacetate).

To profile metabolite abundance changes in the same sample cohorts, we performed widely-targeted metabolomics analysis. A total of 568 metabolites were successfully detected (**Supplementary Table 4**). Among these, 35 organic acids and their derivatives constituted the most abundant class (26.52%), followed by 34 terpenoids (25.76%) and 23 phenolic acids and their derivatives (17.42%) (**Figure 2A**). Partial least squares-discriminant analysis (PLS-DA) was employed to extract the principal features of the metabolite expression matrix (**Figure 2B**). The first two PLS-DA components explained 13.1% and 9.59% of the total variance, respectively. In the two-dimensional score plot, samples from Group D and Group K were distinctly separated. More importantly, within each line, the samples exhibited a continuous and non-overlapping trajectory that strictly followed the developmental sequence (from stage 1 to 9). This clustering pattern indicates that both genotype and developmental stage contributed independently to metabolic profile variation.

Differentially abundant metabolites (DAMs) identified between the two lines at different developmental stages were annotated and classified using KEGG. The DAMs were primarily concentrated in “Amino acid metabolism,” “Carbohydrate metabolism,” and “Metabolism of cofactors and vitamins,” with the differences being most pronounced in the three later developmental stages (S6, S8, and S9) (**Figure 2C**). We focused on the carbohydrate metabolism pathway pertinent to root sugar content (**Figure 2D**). A heatmap of DAM abundances captured a clear signal of directional carbohydrate reorganization. The high-sugar line (Group K) exhibited a highly significant enrichment of core soluble sugars during the later developmental stages (K6 to K9). Among the carbohydrate-related DAMs, raffinose and galactinol showed significant differences between the two lines at specific developmental stages (VIP > 1, p < 0.05), while sucrose and trehalose did not meet the DAM criteria across all stages examined. In stark contrast, key carbon-nitrogen intermediates such as L-glutamine, succinic acid, and L-malic acid showed significant decreases in late developmental stages.

### Unsupervised clustering of temporal transcriptional trajectories and enrichment of key metabolic pathways

**Figure 3:**
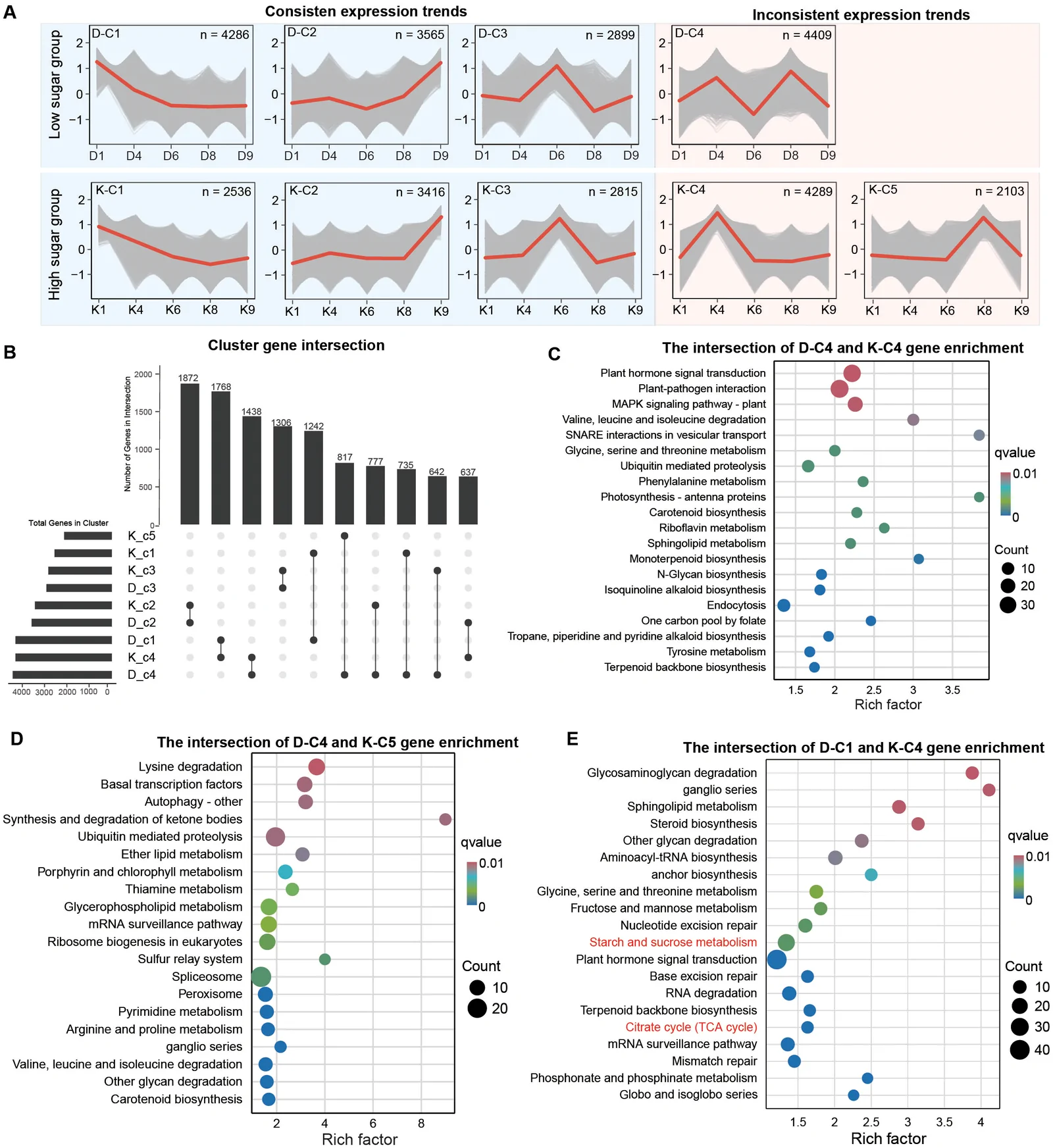
Temporal programming of metabolic priority shifts via differential gene expression trajectories. **(A)** Mfuzz soft clustering revealing conserved and line-specific expression patterns. Red lines indicate cluster centroids; gray shading indicates 95% confidence intervals. **(B)** UpSet intersection analysis showing shared and unique genes between expression clusters. **(C–E)** KEGG pathway enrichment analysis of genes with opposing expression trends between lines, highlighting enrichment in primary carbon and nitrogen metabolism.

To identify gene clusters with distinct temporal expression patterns, we performed time-series clustering of all DEGs using the Mfuzz algorithm. DEGs from Group D and Group K were each partitioned into multiple clusters based on expression trajectory similarity (**Figure 3A**). The two lines shared only three common expression trends: genes that were progressively up- or downregulated throughout development, and genes specifically upregulated at stage 6. In Group D, 4,409 genes were suppressed at stage 6 (D-C4), a trend absent in Group K. Instead, Group K featured two unique gene clusters with specific upregulation at stage 4 (K-C4, 4,289 genes) and stage 8 (K-C5, 2,103 genes). This highlights substantial differences in the developmental progression and gene expression programs between the two lines. An UpSet plot analysis of these clusters revealed that clusters with similar trends largely shared the most genes; clusters C1, C2, and C3 shared 1,242, 1,872, and 1,306 genes, respectively, representing conserved expression patterns in root development (**Figure 3B**). Among the line-specific clusters, K-C4 shared 1,768 and 1,438 genes with D-C1 and D-C4, respectively, while K-C5 shared 817 genes primarily with D-C4. These shared genes exhibited completely opposite expression trends between the two lines.

By analyzing the intersections of gene sets with consistent and inconsistent expression trajectories between the lines, we focused on gene sets that had a large number of overlapping genes but opposing expression patterns. KEGG pathway enrichment analysis showed that genes shared among clusters D-C4, K-C4, and K-C5 were primarily enriched in pathways such as “Plant-pathogen interaction,” “Lysine degradation,” and “Ubiquitin mediated proteolysis” (**Figures 3C, D**). In contrast, the functions of genes overlapping between D-C1 and K-C4 were highly and specifically concentrated in core carbohydrate metabolism and signaling pathways, including “Starch and sucrose metabolism” (q-value < 0.01), “TCA cycle,” and “Plant hormone signal transduction” (**Figure 3E**). The expression of these genes continuously decreased in Group D during the early stages but was activated much earlier in Group K, suggesting that activation of genes in carbohydrate metabolism pathways occurs earlier in the high-sugar line than in the high-yield line.

### Construction of a weighted gene co-expression network and identification of core regulatory hubs

**Figure 4:**
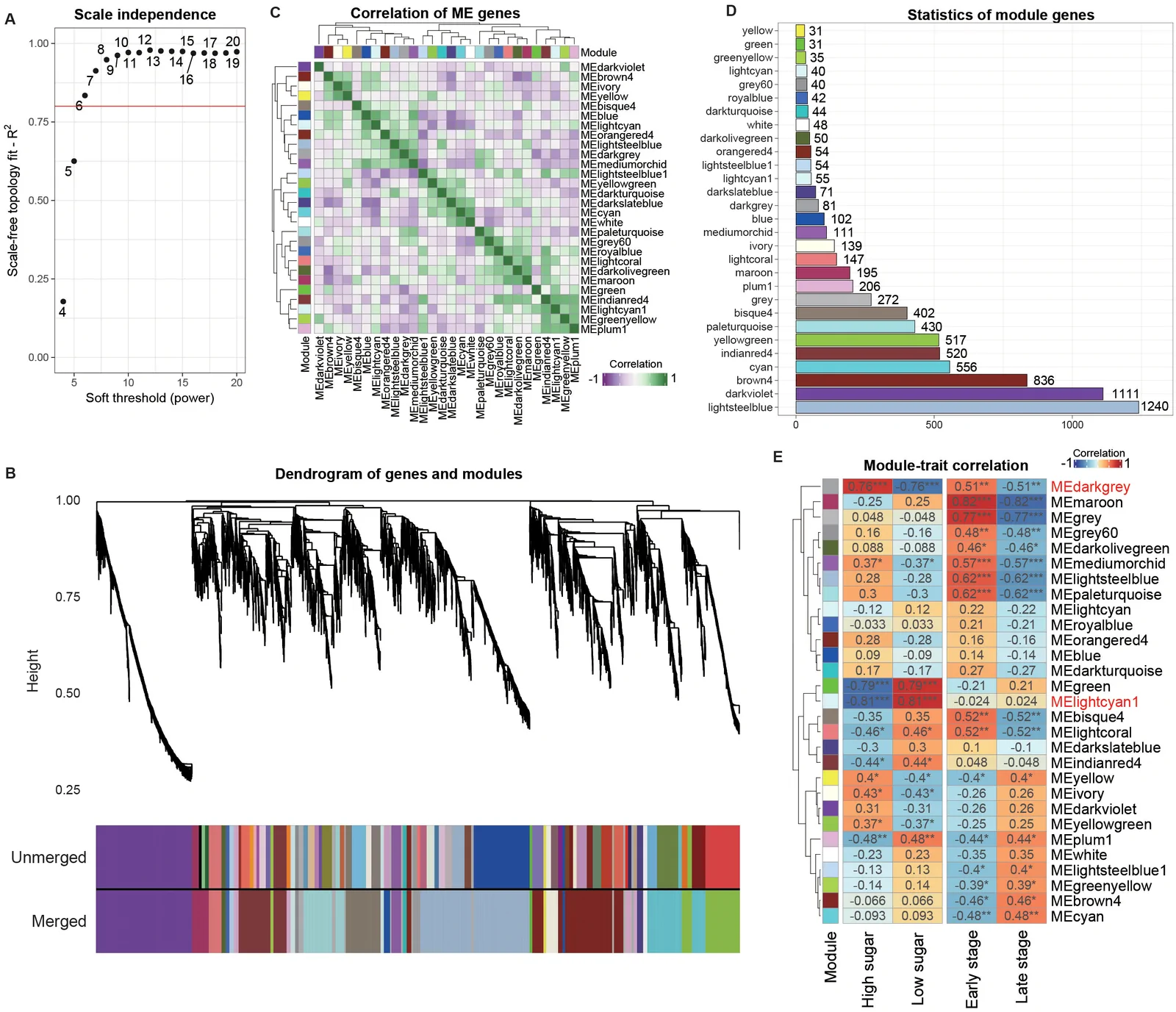
Weighted gene co-expression network analysis (WGCNA) identifies trait-associated regulatory modules. **(A)** Scale-free topology fitting index as a function of soft-thresholding power (β = 6 selected, R² > 0.8). **(B)** Hierarchical clustering dendrogram and module assignment. **(C)** Module-module correlation heatmap based on eigengenes. **(D)** Gene counts per co-expression module. **(E)** Module-trait correlation matrix identifying the Darkgrey module (high-sugar associated, r = 0.76) and Lightcyan1 module (high-yield associated, r = 0.81).

To characterize gene co-expression modules associated with sugar content and root yield, we performed weighted gene co-expression network analysis (WGCNA). Based on a scale-free topology assessment, the optimal soft-thresholding power was set to 6 (fit index R² > 0.8) (**Figure 4A**). Hierarchical clustering of co-expressed genes identified 29 color-coded modules (**Figure 4B**). The low correlation between module eigengenes indicated a clear and distinct partitioning of the modules (**Figure 4C**). The number of genes per module varied widely; for example, the lightsteelblue module contained 1,240 genes, whereas the yellow and green modules contained only 31 genes each (**Figure 4D**). A heatmap of Pearson correlations between module eigengenes and phenotypic traits identified modules significantly associated with sugar content and root yield (**Figure 4E**). The Darkgrey module (81 genes) was significantly correlated with the high-sugar trait (r = 0.76, p < 0.001, FDR < 0.001) and positively correlated with early development (r = 0.51). The Lightcyan1 module (55 genes) showed a significant positive correlation with the low-sugar, high-yield phenotype (r = 0.81, p < 0.001, FDR < 0.001).

### Co-expression network analysis of core sugar metabolism modules

**Figure 5:**
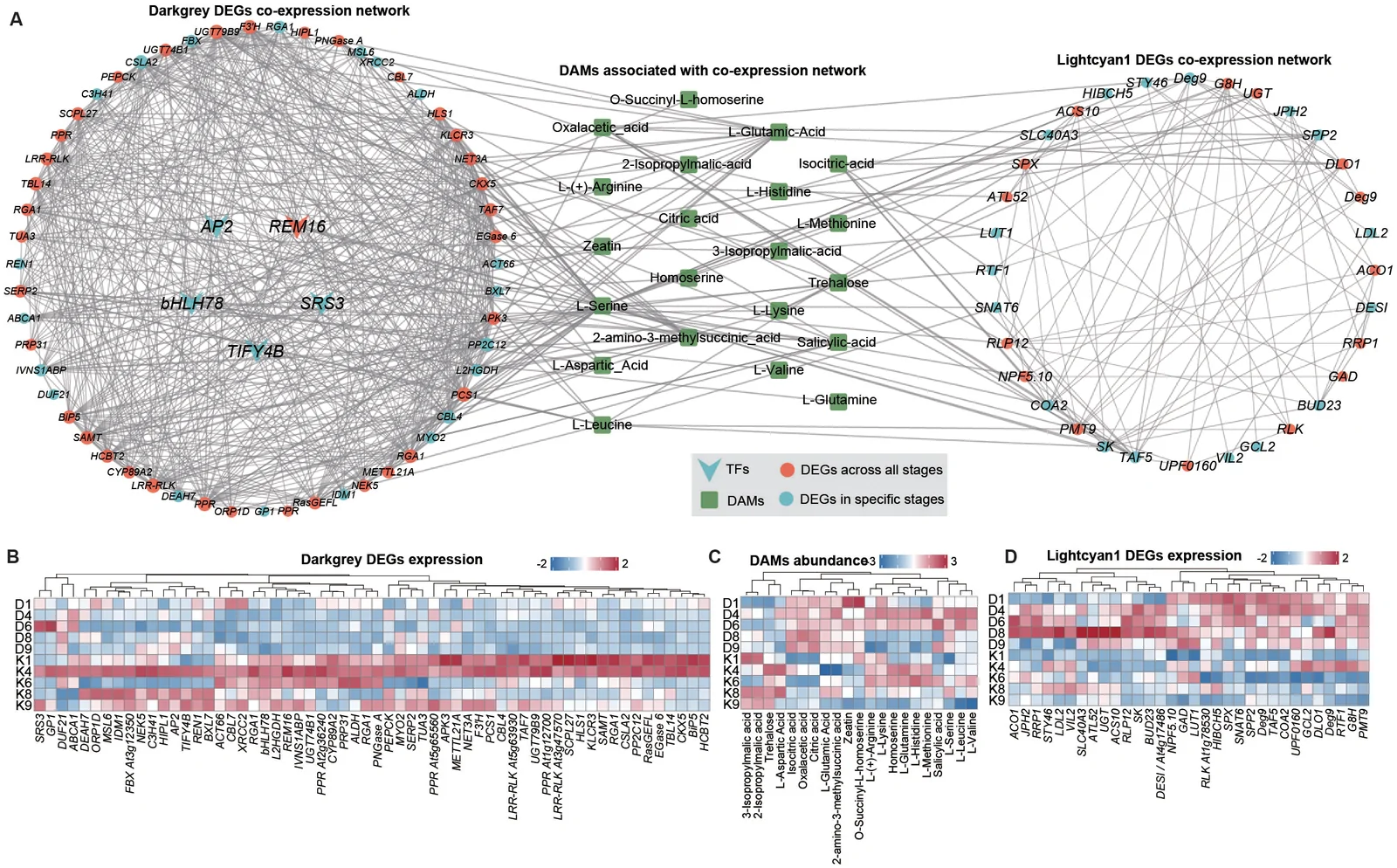
Regulatory network architecture associated with differential carbon allocation. **(A)** Co-expression networks of Darkgrey (left, high-sugar) and Lightcyan1 (right, high-yield) modules. Red nodes: hub transcription factors; light blue: structural genes; green: correlated metabolites. **(B–D)** Expression heatmaps of DEGs and DAMs associated with each module across developmental stages.

Based on intramodular connectivity (weight > 0.5) and gene-metabolite correlations, we reconstructed the gene-metabolite interaction networks for the core modules (**Figure 5A**). The Darkgrey regulatory network comprised 5 transcription factors and 55 co-expressed genes. Among them, 35 were differentially expressed between the two lines during both early (stages 1, 4) and late (stages 6, 8, 9) development. Core transcription factors, including *AP2*, *bHLH78*, *REM16*, *SRS3*, and *TIFY4b*, occupied central hub positions in the network. These TFs are known to be associated with plant organ maturation, auxin/JA signaling, and secondary metabolism regulation. The co-expressed genes in the Darkgrey network included key structural genes related to primary carbohydrate turnover and cell wall remodeling, such as phosphoenolpyruvate carboxykinase (*PEPCK*), UDP-glycosyltransferases (*UGT79B9*, *UGT74B1*), cellulose synthase-like A2 (*CSLA2*), and beta-D-xylosidase 7 (*BXL7*). The Lightcyan1 network contained 32 co-expressed genes. Although no canonical TFs were identified as hubs in this network, its node genes were highly enriched in pathways related to substrate transmembrane transport and primary metabolism. These included rate-limiting enzymes and transporters critical for sucrose synthesis and transport, such as sucrose-phosphate phosphatase 2 (*SPP2*), glutamate decarboxylase (*GAD*), sodium-coupled neutral amino acid transporter 6 (*SNAT6*), and a nitrate/peptide transporter (*NPF5.10*).

The expression of genes in these two networks was tightly correlated with the abundance of 21 metabolites. As shown in the expression heatmap (**Figures 5B–D**), although timing differed, genes in the Darkgrey network were predominantly highly expressed in Group K, whereas those in the Lightcyan1 network were primarily highly expressed in Group D. Among the correlated metabolites, compatible solutes like trehalose were highly accumulated in Group K roots. Conversely, key TCA cycle intermediates such as citric acid, isocitric acid, and oxaloacetic acid, as well as major free amino acids like L-glutamine, L-glutamic acid, and L-arginine, were found at significantly lower levels in Group K. These metabolites are fundamental substrates for aerobic respiration and primary nitrogen assimilation. In contrast, the low-sugar, high-yield line (Group D) maintained a high metabolic flux through pathways involving L-glutamine, oxaloacetate, and malate derivatives (e.g., 2-isopropylmalate). These coordinated patterns suggest that Group K shows reduced expression of primary metabolic genes alongside higher sucrose and osmoprotectant levels during late development, while Group D maintains higher expression of TCA cycle and nitrogen assimilation genes alongside elevated organic acid and amino acid levels.

### Reconstruction of molecular pathways for synergistic carbon-nitrogen metabolism

**Figure 6:**
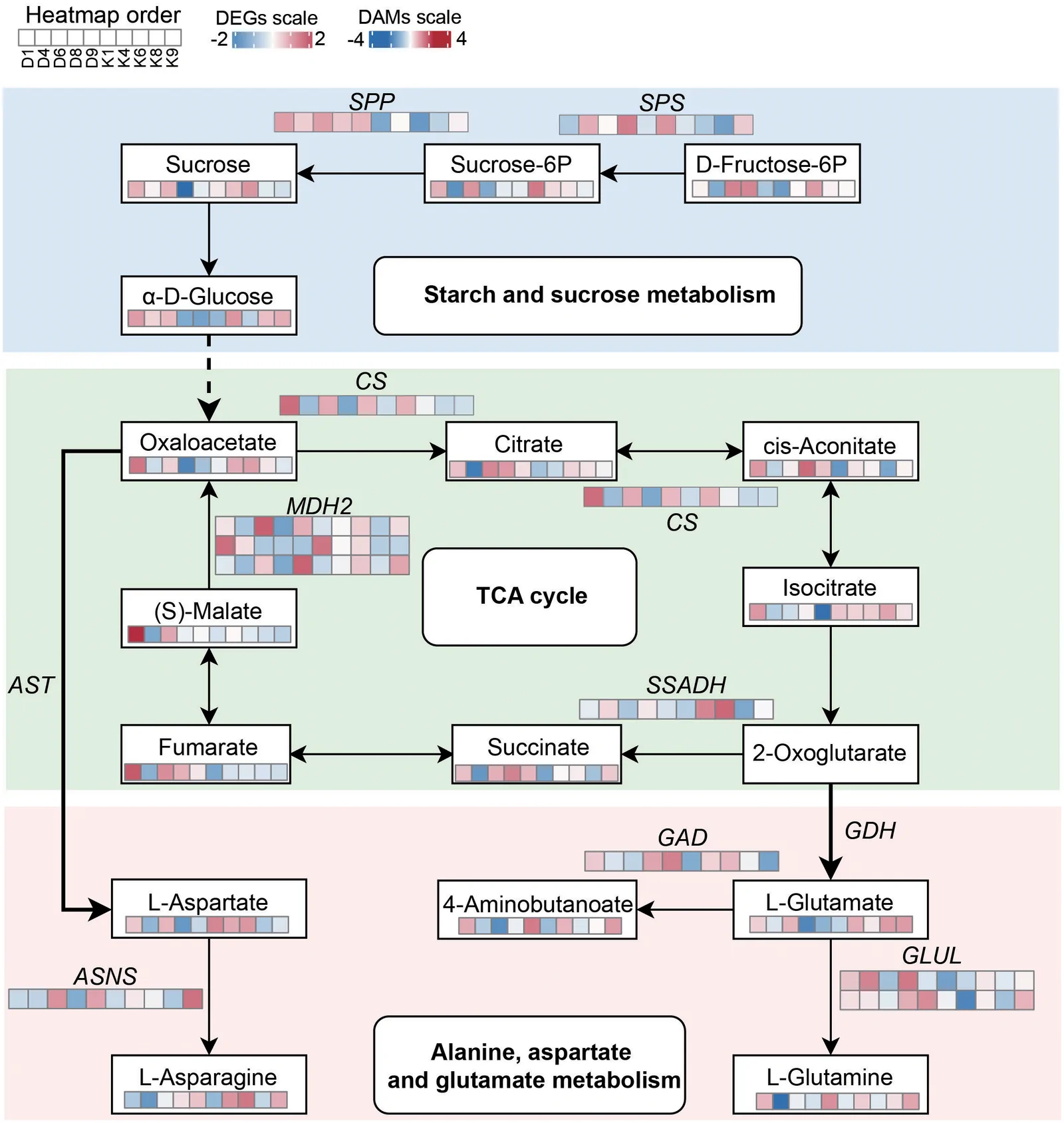
Integrated transcriptomic and metabolomic analysis reveals contrasting carbon allocation strategies. Schematic representation of starch/sucrose metabolism, TCA cycle, and amino acid metabolism pathways. Adjacent heatmaps display relative expression of enzymatic genes (DEGs) and abundance of metabolites (DAMs) across ten samples. Color scales: blue (low) to red (high). Group D maintains active carbon consumption (high *CS*, *ACLY*, *MDH2*, *GLUL*), while Group K exhibits attenuated primary metabolism and enhanced sucrose storage.

By integrating structural genes and differential metabolites from the co-expression networks, we examined expression and abundance patterns across key metabolic pathways in beet taproots, focusing on starch and sucrose metabolism, the TCA cycle, and amino acid metabolism (**Figure 6**). The results revealed that key rate-limiting enzymes in the upstream portion of carbohydrate metabolism—sucrose biosynthesis—exhibited line-specific temporal expression patterns. The *Sucrose Phosphate Synthase (SPS)* gene showed periodic expression peaks in the mid-to-late stages (D4 and D8) in Group D (FPKM values of 40.47 and 47.42, respectively). In Group K, *SPS* transcriptional activation occurred very early (K1, FPKM = 44.07) and experienced a second resurgence before harvest (K9, 36.58). Notably, the *Sucrose Phosphate Phosphatase (SPP)* gene, which catalyzes the final dephosphorylation step, maintained a consistently high constitutive expression level throughout development in Group D (FPKM range: 28.73-34.09), whereas its abundance was relatively lower and more variable in Group K (6.50-24.76).

Despite the higher transcriptional background for precursor synthesis enzymes in Group D, its absolute sucrose accumulation pool showed a significant decline in the late stage (D8, relative abundance dropped to 1.61). Conversely, Group K not only maintained a stable supply of substrates like D-fructose-6-phosphate but also reached a peak sucrose abundance in the mid-stage (K6, 1.87), which remained at a higher and more stable level than in Group D throughout the mid-to-late period. This demonstrates that the establishment of the high-sugar phenotype is not merely dependent on the elevated expression of a single synthesis enzyme but stems from the strict limitation of pathways that consume the net sucrose pool.

The partitioning of carbon skeletons into the TCA cycle was regulated by the spatiotemporal differences in entry-point enzymes. *Citrate Synthase (CS)*, which catalyzes the condensation of acetyl-CoA and oxaloacetate, showed significantly higher expression in Group D at several key time points (D1, D6, D9; FPKM 23.00-29.12) compared to Group K (15.34-18.00). In concert with this, *ATP-Citrate Lyase (ACLY)* exhibited an extremely high, specific transcriptional burst at the initial stage in Group D (D1, FPKM up to 114.98), whereas its peak in Group K occurred later and was weaker (K4, 36.43). At the end of the cycle, *Malate Dehydrogenase (MDH2)*, responsible for converting malate to oxaloacetate, was highly transcribed in both lines throughout all stages, with Group D reaching exceptionally high levels at D6 and D9 (336.69 and 274.88, respectively). The active state of these primary respiratory network genes was directly reflected in the steady-state concentrations of their intermediate metabolites: the pool sizes of citrate, cis-aconitate, and early-stage oxaloacetate were consistently higher in Group D, confirming that the low-sugar, high-yield line maintains a higher flux of basal respiratory carbon turnover to meet the energetic demands of tissue construction.

At the intersection of carbon and nitrogen metabolism, the amino acid synthesis and GABA shunt pathways displayed the most dramatic developmental reprogramming. *Glutamine Synthetase (GLUL)* and *Glutamate Decarboxylase (GAD)* maintained significantly higher expression levels in Group D during the early and mid-stages compared to Group K. The sustained high expression of these key nitrogen assimilation enzymes enabled Group D to continuously pump ample carbon skeletons from the TCA cycle into the L-glutamine and 4-aminobutanoate pools. These were then channeled into structural biosynthesis networks to satisfy the immense biomass requirements for the continuous expansion of the vascular cambium. Together, these patterns indicate that Group D maintains sustained activity of TCA cycle and nitrogen assimilation pathways throughout development, whereas Group K shows progressive reduction in these pathways during later stages, coinciding with elevated sucrose and osmoprotectant accumulation.

### qRT-PCR validation of core genes

**Figure 7:**
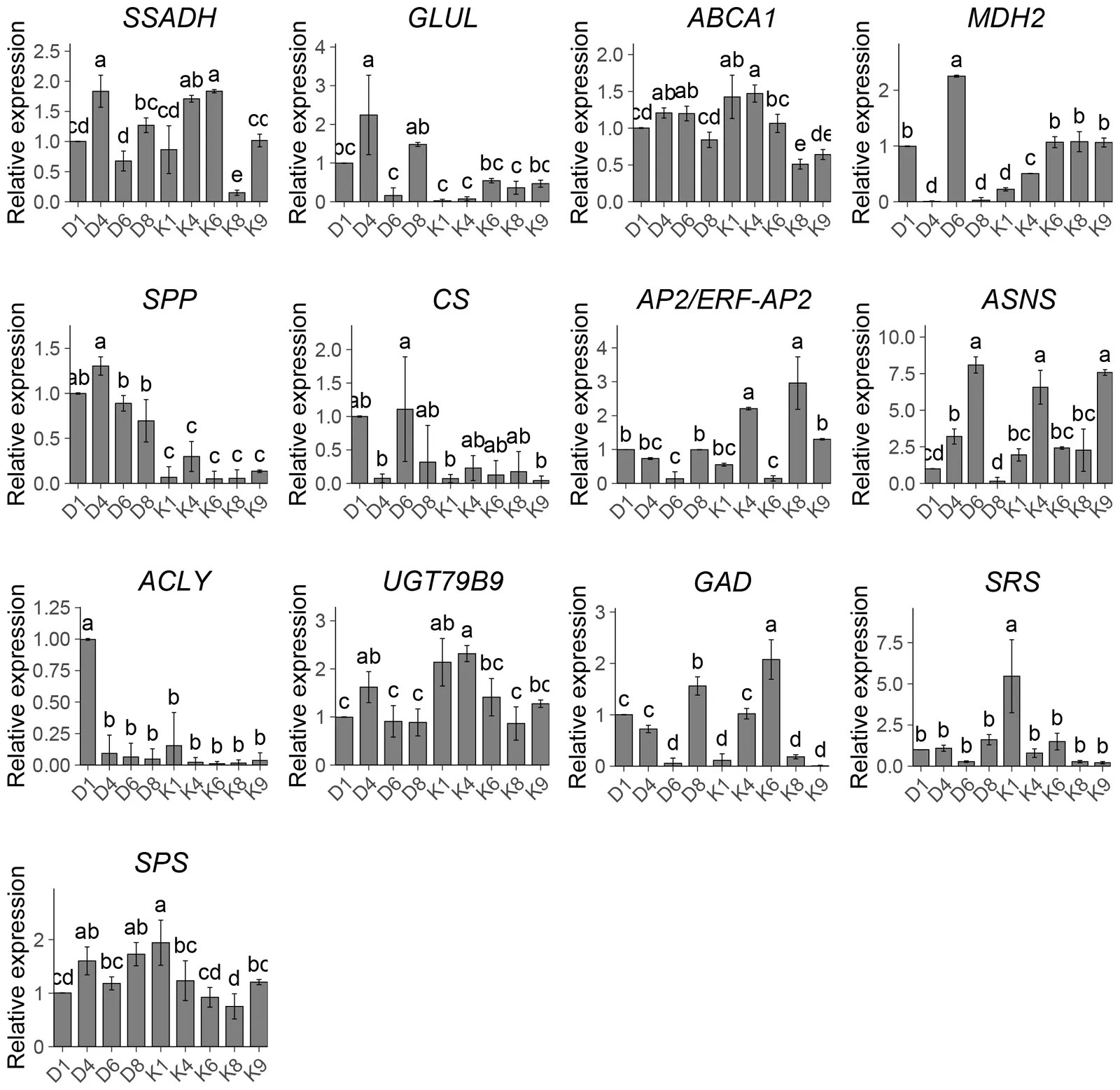
Quantitative RT-PCR validation of RNA-seq data. Expression patterns of representative genes involved in TCA cycle and carbon consumption, nitrogen assimilation, and candidate regulatory hubs. Data represent mean ± SD (n=3 biological replicates). Different lowercase letters indicate significant differences among samples (P < 0.05, one-way ANOVA with Tukey’s test). Pearson correlation between RNA-seq and qRT-PCR results ranged from 0.89 to 0.96.

To validate the RNA-seq results, representative DEGs from the regulatory and metabolic pathways identified above were selected for qRT-PCR analysis (**Figure 7**). The gene expression profiles showed distinctly divergent temporal trends between the two lines. Specifically, genes driving the TCA cycle and early carbon-consuming pathways, such as *ACLY*, *MDH2*, *CS*, and *SPP*, exhibited pronounced expression peaks or sustained higher expression primarily in the low-sugar line (Group D). For instance, *ACLY* showed a massive initial burst at stage D1, while *MDH2* and *CS* spiked significantly at D6. This corroborates the observation that Group D actively diverts carbon flux toward basal respiration and biomass synthesis. In contrast, key transcription factors and structural genes identified in the high-sugar regulatory modules, including *SRS*, *AP2/ERF-AP2*, and *UGT79B9*, showed specific and strong upregulation in the high-sugar line. SRS displayed a striking early transcriptional activation at K1, and *AP2/ERF-AP2* peaked at K4 and K8, indicating an early and distinct transcriptional reprogramming to prime sucrose storage in Group K. The expression trends determined by qRT-PCR were consistent with RNA-seq results across all developmental stages, with Pearson correlation coefficients between the two methods ranging from 0.89 to 0.96. These results confirm the reliability of the RNA-seq quantification data used in this study.

## Discussion

The sugar beet root, as a heterotrophic sink organ, depends on an energy-dependent process of carbon import, metabolism, and vacuolar sequestration. A conventional view posits that high-level accumulation of a target metabolite is directly driven by the robust expression of its biosynthetic genes (Aničić et al., 2021). However, the present findings suggest a different pattern. Counterintuitively, the low-sugar, high-yield line (Group D) exhibited higher transcript abundances for key sucrose synthesis genes, including Sucrose Phosphate Synthase (*SPS*) and Sucrose Phosphate Phosphatase (*SPP*), during the late developmental stages. Despite this, its final sucrose pool underwent significant depletion. This suggests that the high-sugar phenotype in beet may reflect suppressed catabolism and reduced metabolic consumption rather than enhanced synthetic capacity.

This strategy of prioritizing catabolic inhibition over synthetic enhancement finds parallels in other root and tuber crops, while contrasting with stem-storing plants. In sugarcane (*Saccharum officinarum* L.), for instance, sucrose accumulation is directly correlated with the expression level of the *SoSPS1* gene, a classic “synthesis-driven” model (Anur et al., 2020). Conversely, for sink organs like potato (*Solanum tuberosum* L.) tubers, which undergo continuous cambial activity, maintaining sink strength is tightly coupled with managing primary metabolism. The downregulation of the vacuolar acid invertase gene *StvacINV1* is essential to prevent a futile cycle of sucrose cleavage during tuber maturation (Yasmeen et al., 2022). Similarly, in sweet potato (*Ipomoea batatas* L.), peak Sucrose Synthase (SuSy) activity aligns with the initial phase of root expansion, where imported sucrose is rapidly cleaved to fuel starch biosynthesis (Cai et al., 2022). Our results share some conceptual parallels with these non-gramineous storage organs. It should be noted, however, that sugar beet taproots develop through anomalous cambial activity producing multiple concentric vascular rings, a structural organization distinct from both potato tubers and sweet potato storage roots. This anatomical difference may influence the nature of sink regulation, and cross-crop comparisons should therefore be interpreted with caution. In Group D, abundant *SPS* and *SPP* transcripts were associated with higher carbon flux into secondary metabolism and structural growth. In Group K, early *SPS* expression was followed by accumulation of compatible solutes including trehalose, raffinose, and galactinol in later stages (K6 to K9). These solutes may contribute to cellular protection under the high osmotic conditions associated with sucrose accumulation (Ghosh et al., 2021), though whether they actively promote sucrose accumulation or represent downstream responses to elevated sucrose levels remains to be clarified. This temporal divergence in gene expression and metabolic outcome indicates that the high-sugar trait is achieved by developmentally shifting the carbon equilibrium towards a state of minimized consumption.

The physical expansion of a storage organ demands significant energetic investment through aerobic respiration and a steady supply of carbon skeletons for nitrogen assimilation (Lawlor, 2002). Our integrated analysis provides evidence on the metabolic basis of the yield-sugar trade-off. The high-yield Group D maintained exceptionally high transcriptional activity of key TCA cycle genes—such as Citrate Synthase (*CS*), ATP-Citrate Lyase (*ACLY*), and Malate Dehydrogenase (*MDH2*)—throughout its development. This was coupled with the sustained high expression of Glutamine Synthetase (*GLUL*) and Glutamate Decarboxylase (*GAD*), corresponding to high flux through L-glutamine, oxaloacetate, and malate-derived pathways. This evidence supports a model where the yield-sugar antagonism arises from the partitioning of carbon skeletons between the TCA cycle for energy production and branch pathways for amino acid synthesis. This suggests that biomass accumulation is associated with sustained allocation of photosynthate toward nitrogen metabolism and respiratory energy production during root development.

It should be noted that the present study focused primarily on transcriptional regulation of primary metabolic pathways and did not directly examine the expression or activity of vacuolar sucrose transporters. BvTST2.1, the tonoplast transporter responsible for active sucrose loading into vacuoles against a concentration gradient, has been established as a key determinant of sugar accumulation in sugar beet (Jung et al., 2015). Differences in BvTST2.1 expression or transport capacity between the two lines may also contribute to their phenotypic divergence, a question that was not addressed in the current analysis. In addition, sugar signaling pathways—including trehalose-6-phosphate signaling and hexose sensing—are known to regulate source-sink carbon allocation in plants and may interact with the transcriptional networks identified here. Future studies integrating transporter-level analysis with the regulatory network described in this work would provide a more complete picture of the mechanisms governing the yield-sugar trade-off.

In stark contrast, the high-sugar Group K executes a transcriptional shutdown of these energy-intensive pathways during its maturation phase. Metabolomic data confirmed this metabolic quiescence, revealing significantly lower pools of TCA intermediates (citrate, isocitrate, oxaloacetate) and major free amino acids (L-glutamate, L-arginine). This regulatory strategy is conserved across diverse plant systems. For example, reducing SlMDH expression in tomato (*Solanum lycopersicum* L.) fruit effectively dampens respiratory flux and redirects carbon to storage pools (Imran et al., 2022). In maize (*Zea mays* L.) kernels, variations in ZmPEPCK2 activity directly dictate the final ratio of starch to storage protein (Yang et al., 2026). Our findings suggest that reduced activity at entry points of the TCA cycle and nitrogen assimilation is associated with the high-sugar phenotype in Group K. This reduction in primary metabolic activity may contribute to preferential sucrose accumulation in Group K.

Co-expression network analysis showed that the Darkgrey module, most significantly associated with the high-sugar trait, was not enriched with structural genes from primary carbon metabolism. Instead, the “Lightcyan1” module, which included key enzymes like SPP2 and GAD, was more active in the low-sugar, high-yield line. This indicates that the high-sugar phenotype is not governed by the extreme upregulation of a single biosynthetic gene. Instead, the Darkgrey module was characterized by a network of hub transcription factors, suggesting that the high-sugar phenotype may be coordinated at the regulatory level rather than through individual structural genes.

Within the Darkgrey module, AP2, bHLH78, REM16, SRS3, and TIFY4b were identified as hub transcription factors with high intramodular connectivity, representing candidates for regulatory roles in the metabolic divergence between the two lines. The AP2/ERF superfamily has established roles in developmental timing and stress responses across plant species (Zhu et al., 2021). The identification of AP2 as a hub gene co-expressed with growth-associated genes in the Darkgrey module suggests a possible involvement in the growth-to-storage transition in sugar beet, though its direct transcriptional targets in this context remain to be determined. bHLH transcription factors have been implicated in metabolic regulation in other species; for example, OsbHLH144 in rice (*Oryza sativa* L.) has been associated with sucrose loading and starch synthesis (Ren et al., 2023). The co-expression of bHLH78 with TCA cycle genes in the Darkgrey module raises the possibility that it may participate in carbon partitioning regulation in sugar beet, though functional evidence for its direct targets is currently lacking. REM16, a B3 domain protein previously associated with developmental regulation (Yu et al., 2020), was also identified as a hub gene; its potential role in sugar beet taproot maturation warrants further investigation.

The identification of TIFY4b, a JAZ family repressor of jasmonate signaling (JA), as a hub gene raises the possibility that hormone signaling may be involved in this metabolic transition. Sugar molecules act as primary signals that deeply crosstalk with hormone networks. The defense hormone JA typically inhibits vegetative growth while activating costly secondary metabolism. In wheat (*Triticum aestivum* L.), TIFY family members regulate cell expansion during grain filling (Xie et al., 2019). By suppressing JA signaling, TIFY4b in the high-sugar beet line may prevent the diversion of carbon to defense-related compounds. Together, the co-expression of these transcription factors with primary metabolic genes is consistent with a model in which the high-sugar phenotype reflects the coordinated activity of multiple regulatory factors, rather than a single structural gene. Functional characterization of these candidates through gene silencing, overexpression, or genome editing will be necessary to establish their individual contributions.

The negative correlation between root yield and sugar content has long constrained simultaneous improvement of both traits in sugar beet. The present findings suggest that this trade-off reflects differential carbon allocation between growth-supporting metabolic pathways and vacuolar sucrose storage during development, rather than variation at a single biosynthetic step. The candidate regulatory factors that identified in this study—particularly the hub transcription factors in the Darkgrey module—may provide a molecular basis for future efforts to modify this balance. Functional characterization of these candidates through genetic studies will be a necessary step before any breeding application can be considered, and the gene-metabolite associations described here offer a starting point for such investigations.

## Conclusion

This study reveals the molecular basis of the yield-sugar antagonism in sugar beet taproots. The high-sugar phenotype appears to reflect suppression of primary metabolic consumption—particularly the TCA cycle and nitrogen assimilation pathways (*CS*, *MDH2*, *GLUL*, *GAD*)—rather than enhanced sucrose biosynthesis. In contrast, the high-yield line maintains high activity of these pathways to support continuous biomass expansion. This difference in pathway activity is associated with lower nitrogenous metabolite levels and higher sucrose and osmoprotectant accumulation (e.g., trehalose) in Group K. This metabolic divergence is accompanied by a co-expression module containing hub transcription factors *AP2*, *bHLH78*, *REM16*, *SRS3*, and *TIFY4b*, suggesting that regulatory rather than structural genetic control may underlie the high-sugar phenotype. These findings identify candidate regulatory that can be targeted to decouple root growth from sucrose accumulation, providing a foundation for improving yield and sugar content simultaneously in sugar beet.

## Data Availability

The raw transcriptome sequencing data covered in this paper has been uploaded to National Center for Biotechnology Information, accession number is PRJCA063297.

## Supplementary material

Supplementary Table 1 The primer list.

Supplementary Table 2 Phenotypic traits of Group K (S72-8) and Group D (DR41K) at harvest.

Supplementary Table 3 RNA-seq data quality control.

Supplementary Table 4 Metabolite detection information.
